# Artificial Intelligence-Driven Wireless Sensing for Health Management

**DOI:** 10.3390/bioengineering12030244

**Published:** 2025-02-27

**Authors:** Merih Deniz Toruner, Victoria Shi, John Sollee, Wen-Chi Hsu, Guangdi Yu, Yu-Wei Dai, Christian Merlo, Karthik Suresh, Zhicheng Jiao, Xuyu Wang, Shiwen Mao, Harrison Bai

**Affiliations:** 1 The Warren Alpert Medical School, Brown University, Providence, RI 02903, USA; 2 School of Medicine, The Johns Hopkins University School of Medicine, Baltimore, MD 21205, USA; 3 Department of Radiology and Radiological Sciences, Johns Hopkins School of Medicine, Baltimore, MD 21205, USA; 4 Department of Medical Imaging and Intervention, Linkou Chang Gung Memorial Hospital, College of Medicine, Chang Gung University, Taoyuan 33302, Taiwan; 5 Department of Diagnostic Radiology, Warren Alpert Medical School of Brown University, Providence, RI 02903, USA; 6 School of Computing and Information Sciences, Florida International University, Miami, FL 33199, USA; 7 Department of Electrical and Computer Engineering, Auburn University, Auburn, AL 36849, USA

**Keywords:** wireless sensing, artificial intelligence, early diagnosis, healthcare monitoring

## Abstract

(1) Background: With technological advancements, the integration of wireless sensing and artificial intelligence (AI) has significant potential for real-time monitoring and intervention. Wireless sensing devices have been applied to various medical areas for early diagnosis, monitoring, and treatment response. This review focuses on the latest advancements in wireless, AI-incorporated methods applied to clinical medicine. (2) Methods: We conducted a comprehensive search in PubMed, IEEEXplore, Embase, and Scopus for articles that describe AI-incorporated wireless sensing devices for clinical applications. We analyzed the strengths and limitations within their respective medical domains, highlighting the value of wireless sensing in precision medicine, and synthesized the literature to provide areas for future work. (3) Results: We identified 10,691 articles and selected 34 that met our inclusion criteria, focusing on real-world validation of wireless sensing. The findings indicate that these technologies demonstrate significant potential in improving diagnosis, treatment monitoring, and disease prevention. Notably, the use of acoustic signals, channel state information, and radar emerged as leading techniques, showing promising results in detecting physiological changes without invasive procedures. (4) Conclusions: This review highlights the role of wireless sensing in clinical care and suggests a growing trend towards integrating these technologies into routine healthcare, particularly patient monitoring and diagnostic support.

## 1. Introduction

In healthcare, chronic diseases such as diabetes, heart disease, and respiratory illness require continuous dynamic monitoring to prevent complications and manage symptoms. Chronic medical conditions currently account for 75% of U.S. healthcare costs, demonstrating the need for reliable at-home health monitoring solutions for personalized care and improved quality of life [1]. Currently, traditional healthcare is not equipped to provide 24/7 oversight to patients, especially outside of clinical environments. To improve health outcomes and facilitate proactive health management, wireless sensing has emerged as a technological frontline, enabling not only dynamic symptom monitoring but also proactive outcome management. In this context, artificial intelligence (AI)-driven wireless sensing has significant potential to transform healthcare monitoring and interventions, making healthcare more accessible and responsive to patient needs.

Over the years, medical researchers have tried to develop patient-centered methods for health monitoring outside clinical settings [2]. Advances in computing power, data processing, and ultra-high-powered microchips have made wireless sensing a promising tool for various applications. Broadly, wireless sensing involves devices that emit and/or receive wireless signals to detect physiological data. Wearable sensors, such as smart watches, are widely used to monitor vital signs like heart rate, respiratory rate, and oxygen saturation [3]. Other applications include ambulatory blood pressure monitoring, mobile cardiac telemetry, and smartphone blood glucose monitoring. 

AI plays a crucial role in healthcare wireless sensing by facilitating data processing, analysis, and interpretation. Through predictive modeling, it identifies trends that enable personalized, proactive care. The growing demand for contactless healthcare solutions, especially post-pandemic, has led to AI applications beyond simple vital sign monitoring, encompassing clinical decision support systems capable of predicting disease exacerbations or life-threatening events. However, the effectiveness of these approaches is limited by patient health literacy, compliance, and the need for cumbersome wearable sensors. These challenges have paved the way for more convenient and reliable contactless methods for ambulatory health monitoring.

Several review articles have explored wireless sensing methods for health monitoring and clinical decision-making. Bhatt et al. focused on AI-incorporated mobile health, specifically smartphone applications and mobile sensors [4]. Similarly, Baig et al. conducted a systematic review of wearable systems, while Kaidi et al. analyzed technical aspects of wireless sensing system designs [5,6]. Other reviews have covered mental health monitoring with wearables, radar-based vital sign detection methods, and contactless sensors in hospitals and home settings [7,8,9].

In this review, we provide a novel and clinically relevant analysis of recent advances in wireless AI-integrated sensing technologies. We focus on studies that evaluate disease-specific wireless sensing applications tested in clinical settings. Unlike previous reviews, we exclude general applications (i.e., vital sign monitoring) of wireless sensing and wearable devices requiring patient input (i.e., smartphone applications). By synthesizing the latest developments in the field, we aim to highlight the limitations of current methods and identify areas for further research, envisioning a future where wireless sensing leads to precise, efficient, and data-driven patient care.

## 2. Materials and Methods

A comprehensive search of the literature was performed by accessing PubMed, IEEEXplore, Embase, and Scopus. The following inclusion criteria were used to identify the most clinically relevant studies: (i) integration of AI for signal processing or data analysis; (ii) designed specifically for diagnosing, prognosing, or monitoring patients with a medical condition or disease; and (iii) tested or validated in real-world clinical scenarios with patients affected by a specific disease, rather than laboratory environments or using healthy subjects mimicking disease states. Simulated conditions were only considered when ethical or logistical constraints prevented real-world testing (e.g., simulated falls in elderly patients). The search was limited to the last ten years (2014 to 2024) to reflect recent advancements in the field. 

Papers focused on general vital sign monitoring without demonstrated healthcare application or on clinical applications of wireless sensing without disease-specific use were excluded. Moreover, given that “wireless sensing” is defined broadly, we restricted this review to studies incorporating radar, WiFi channel state information (CSI), radio frequency identification (RFID), and acoustic signals. Articles from conference proceedings were excluded due to insufficient information for study eligibility assessment.

The search strategy included the following keywords and combinations: “(“wireless sens*” OR “wireless” OR “wireless devic*” OR “wireless technolog*” OR “remote sensing technolog*” OR “wearable electronic device*”) AND (“healthcare” OR “health” OR “medical” OR “monitor*” OR “diagnostic” OR “prognostic” OR “therapeutic”) AND (“radar” OR “rfid” OR “radio frequency identification device” OR “radio frequency” OR “acoustic” OR “csi” OR “channel state information”)”. 

Covidence was used as the primary screening tool [10]. Duplicates were manually removed during screening. Initial title and abstract screening were conducted by M.D.T., with article eligibility independently confirmed by V.S. Secondary abstract screening was independently performed by M.D.T and V.S. Conflicts were resolved throughout the screening process through mutual agreement and/or consultation with H.B.

## 3. A Brief Overview of Wireless Sensing Technology

Wireless sensing refers to technology capable of emitting and receiving signals without the need for wearable hardware. The most utilized wireless signals in healthcare are radar, WiFi CSI, RFID, and acoustic signals. The technical considerations of these wireless signals have been extensively covered by one of the co-authors (X.W.) in prior work (Table 1) [11].

### 3.1. Radar

Radar signals are utilized for a wide range of healthcare applications, including movement detection, vital sign monitoring, and sleep apnea assessment. The three primary radar signal techniques are CW, FMCW, and IR-UWB. Radar devices emit signals that bounce off a target, and the receiver detects changes in frequency or phase shifts in the reflected signal. The key advantage of radar technology lies in its large bandwidth (e.g., FMCW and IR-UWB) and high directional performance, which can be further enhanced using directional antennas to amplify signal strength. However, limitations include their relatively higher cost, as available devices are often expensive and not yet widely accessible for broader clinical use.

### 3.2. CSI

CSI relies on WiFi, using OFDM at the physical layer, where the amplitude or phase of the CSI is used as a sensing feature. Many standard WiFi network interface cards can be modified to extract CSI, which captures propagation effects such as shadowing, power distortion, multipath, and reflections. The main advantages of CSI include its high resolution and relatively low cost. However, environmental factors can impact its accuracy.

Most healthcare applications of CSI use amplitude or phase difference data for monitoring vital signs. For instance, CSI amplitude has been used to monitor respiration, sleep posture [12], and heart rate [13]. While CSI phase data cannot directly detect vital signs, various techniques, such as phase difference, have been developed to overcome this limitation and improve its utility in healthcare monitoring [14,15].

### 3.3. RFID

Originally designed for identifying objects or people, RFID has evolved into a powerful tool over the past few decades for obtaining and detecting relevant health information, such as heart rate variability [16], sleep apnea and changes in respiration [17,18], and body temperature [19]. RFID relies on CW signals, with phase data typically used for sensing. In practice, the patient wears a “tag” or “smart label”, and the distance between the tag and an antenna is measured using phase data, allowing the system to capture physiological information such as chest wall motion. 

RFID offers several advantages, including low cost, easy integration into existing infrastructure, long battery life, low maintenance, and high directional performance [20]. However, it is also subject to limitations, such as susceptibility to channel hopping and variability in performance due to environmental factors.

### 3.4. Acoustic Sensing

Acoustic sensing, both passive and active, has emerged as a powerful method for extracting clinically relevant information. In passive sensing, microphones capture sounds from the surrounding environment and data processing techniques are applied to analyze the captured sound. For instance, smartphone microphones have been used to record nocturnal breathing sounds for respiration analysis [21]. In active sensing, a device generates sonar signals that bounce off the target and are reflected to the microphone. The system then assesses differences in acoustic phase and/or distance. Acoustic sensing offers the advantages of high resolution and convenience. However, it is susceptible to background noise and has a small effective coverage range, limiting its application in certain environments.

## 4. Results

### 4.1. Characteristics of Individual Studies

**Figure 1:**
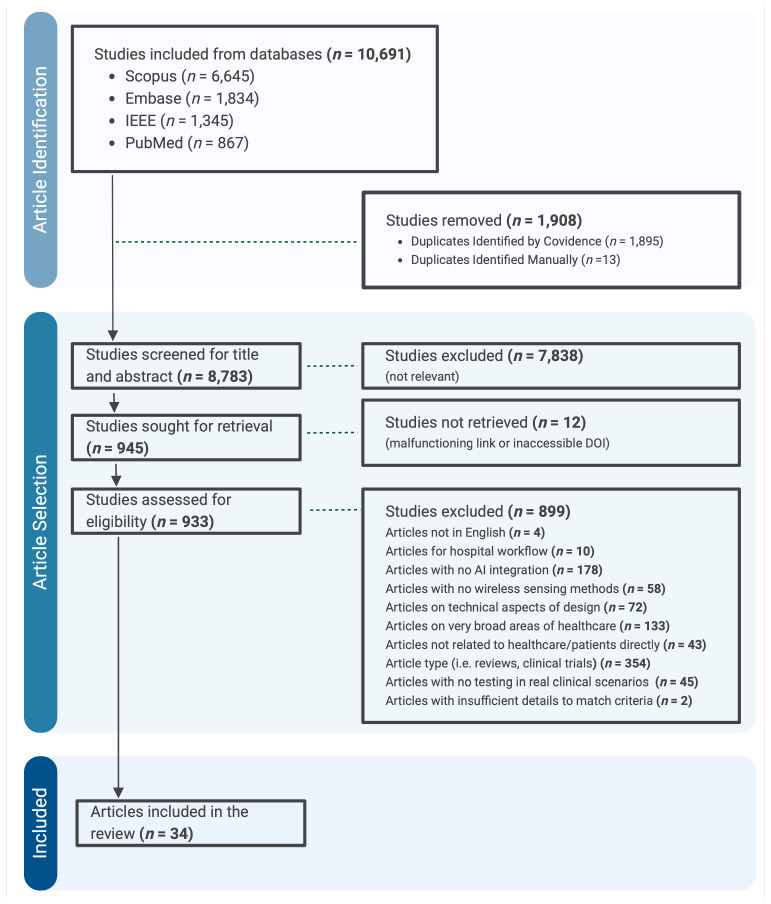
Literature search and selection process in this study.

The search identified 10,691 articles from Scopus, Embase, IEEEXplore, and PubMed. After removing duplicates and non-relevant studies, 945 articles were selected for further evaluation. Of these, 34 articles met the inclusion criteria (Figure 1) and were analyzed within the context of their respective medical fields (Table 2).

**Figure 2:**
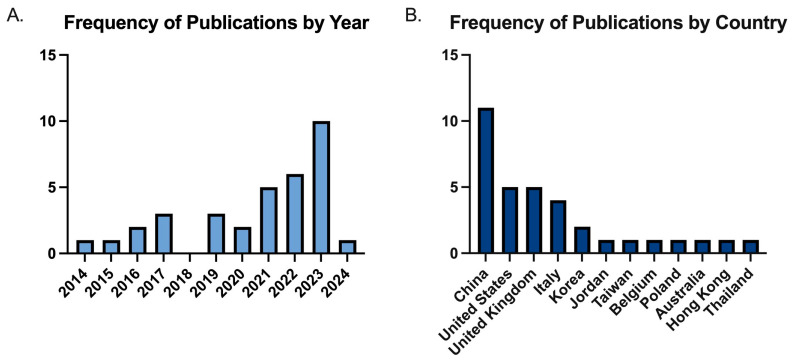
Study characteristics. (**A**) Distributions of publication year of the studies. (**B**) Distributions of country of origin of the first author in included studies.

Although the literature search covered articles from 2014 to 2024, most publications (n = 22) appeared after 2020, compared to 12 published in 2020 or before (n = 12) (Figure 2A). Regarding the primary author’s research institution, the majority of studies originated from China (11, 32.4%), the United States (5, 14.7%), and the United Kingdom (5, 14.7%), followed by Italy (4, 11.8%), Korea (2, 5.9%), Jordan (1, 2.9%), Taiwan (1, 2.9%), Belgium (1, 2.9%), Poland (1, 2.9%), Australia (1, 2.9%), Hong Kong (1, 2.9%), and Thailand (1, 2.9%) (Figure 2B). 

**Figure 3:**
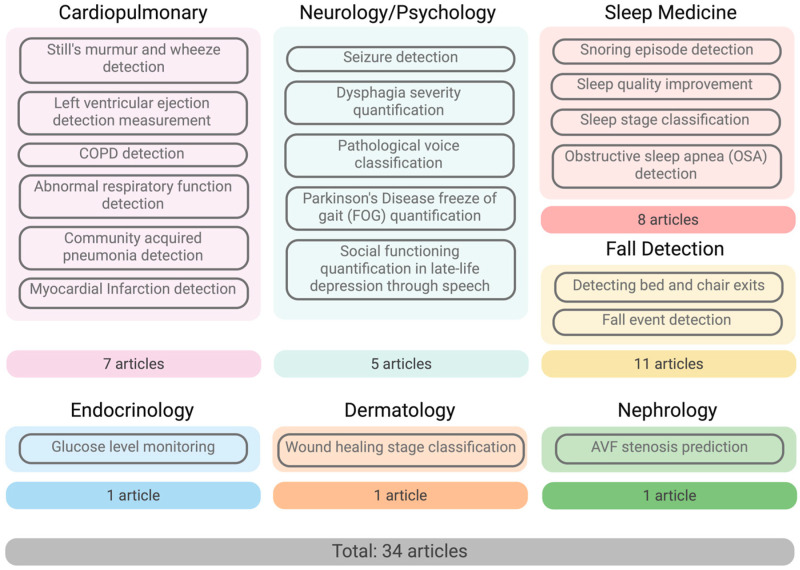
Medical areas of selected studies.

In terms of methodology, 15 of the 34 articles employed deep learning, 16 used machine learning, and 3 utilized both approaches. The majority of the articles were considered clinical or conceptual validation studies for validation of the technology; only a select few articles were feasibility or pilot patient studies or validated through an external dataset (Table 2). The most frequently used wireless sensing method was acoustic (35.3%), followed by CSI (29.4%), radar (23.5%), RFID (5.9%), RF near-infrared spectrometry (2.9%), and mechano-acoustic (2.9%). The articles were categorized into seven distinct areas: fall detection (32.4%), sleep medicine (23.5%), cardiopulmonary (20.6%), neurology and psychology (14.7%), endocrinology (2.9%), dermatology (2.9%), and nephrology (2.9%) (Figure 3).

According to our inclusion criteria, we selected studies that validated their wireless sensing methods using real-world clinical scenarios involving patients with specific medical conditions. Most studies successfully utilized patient data with the targeted disease [22,23,24,26,27,28,30,31,32,33,42,55,56]. For fall detection studies in the geriatric population, however, healthy volunteers were often recruited to simulate falls due to ethical concerns related to patient safety [43,44,45,46,47,48,49,50,52,53]. An exception was Torres et al., who were able to recruit hospitalized patients in a geriatric evaluation and management unit to detect bed and chair exits, aiming to prevent falls in hospital settings [42]. Of note, many studies focused on wireless sensing methods in sleep medicine utilized healthy volunteers, as a specific pathology was not always required [34,35,36,37,38,39,40,41,51]. These studies typically investigated snoring episodes, monitored sleep quality, and classified sleep stages.

Recruitment of participants was a common bottleneck for most studies included in this review due to the stringent protocols required. The majority of studies (22, 65%) had fewer than 50 participants. Six studies had participant numbers ranging from 50 to 100 [23,26,27,33,36,43], while six studies recruited more than 100 participants [22,24,25,28,31,35]. Some studies utilized pre-existing datasets to increase their participant numbers [24,25,31,43,50]. These included hospital voice recordings for asthma attack detection, combined datasets of healthy and pathological voice samples, and the Device-Free Human Activity Recognition and Monitoring System (DARMS) dataset, which consists of CSI signals [51].

### 4.2. Findings of Wireless Sensing Studies in Personal Health

#### 4.2.1. Cardiopulmonary

Wireless monitoring in cardiology and pulmonology has the potential for earlier detection of critical events such as myocardial infarction, and thus offers benefits of minimizing further complications, facilitating early management, extending patient care to the home, and reducing hospital visits and costs. Currently, wireless sensing for early detection and disease monitoring in cardiology includes efforts to detect myocardial infarction and automatic prediction of left ventricular ejection fraction, such as the Health-Radio model, to reduce time to treatment [23,27]. AI-powered digital stethoscopes have also been developed to support disease diagnosis, provide active noise cancelation, and enhance telehealth services. Examples include the StethAid to detect Still’s murmur and wheezes in pediatric patients and a deep learning-based bilateral pulmonary audio-auxiliary model for detecting community-acquired pneumonia [22,28]. Other studies for pulmonary function using wireless sensing have focused on detecting chronic obstructive pulmonary disease, asthma, and other pulmonary diseases [24,25,26].

#### 4.2.2. Neurology and Psychiatry

With many neurological and psychiatric diagnoses relying on subjective observations or self-reports, wireless sensing has been utilized to obtain automated and objective measurements of movement and speech. Various wireless sensing applications have been explored in movement disorders, swallowing and speech dysfunctions, and seizure detection. Wireless sensing has been extensively applied to Parkinson’s disease, allowing remote monitoring of motor and non-motor symptoms, objective analysis of gait parameters, and seizure detection [32,57,58,59]. Such non-invasive detection and monitoring approaches include a CNN-based model, WiFreeze, to quantify and detect freezing of gait in Parkinson’s, and an SVM model for seizure detection using video accelerometry and radar sensing data [29,32]. For other applications, such as in speech detection and swallowing impairment, acoustic wireless sensing has primarily been utilized [30,31,33]. Wireless sensing applications also extend to psychiatry, with one study in our search focusing on detecting speech as a marker for social functioning in late-life depression using acoustic sensing [33]. These studies highlight the potential of wireless sensing technologies to provide objective and accurate assessments of neurological and psychiatric disorders, enabling more precise monitoring and early intervention.

#### 4.2.3. Sleep Medicine

Wireless sensing offers a non-invasive approach for real-time sleep monitoring without needing intrusive equipment that can otherwise interfere with sleep quality. Several sleep monitoring devices for sleep detection or sleep stage classification have been developed, including the Sleepy system, WiFi-Sleep, and an impulse-radio ultra-wideband radar system by Kwon et al. [36,37,40]. Wireless sensing, primarily acoustic sensing, has been used to detect and classify respiratory-related sleep events such as snoring and sleep apnea with high accuracies [34,38,41]. There has also been a growing interest in understanding and improving sleep quality. Gui et al. proposed a WiFi CSI-based system with a CNN model to quantify and analyze sleep turnover events and breathing rates, and Nguyen et al. aimed to not only capture real-time vital signs and sleep posture information and predict sleep stages but also provide auditory stimuli feedback to improve sleep quality [35,39].

#### 4.2.4. Fall Detection for Geriatrics

All included articles in geriatrics were for fall detection. Falls are a significant concern in the geriatric population, with more than one in four older adults experiencing a fall globally [60]. Current fall detection strategies include medication management, gait and balance exercises, patient room hazard assessment, and fall risk assessment tools like STRATIFY [61,62]. However, these approaches are limited by a lack of continuous monitoring and accessibility, and thus wireless sensors offer a promising solution for continuous non-intrusive monitoring, providing rapid assistance and real-time alerts. Most of the fall detection studies either used radar sensing [43,44,47,53] or WiFi CSI sensing [45,46,48,49,50,52] technologies, with one study utilizing RFID [42]. Most of these studies focused on classifying general daily activity, including falls of elderly individuals, with few approaches incorporating more novel features such as post-fall localization, mobile app integration for alerts and management actions, and identification of critical life-threatening falls [47,49,53].

#### 4.2.5. Endocrinology

Wireless sensing can be used to track key physiological parameters, such as blood glucose and insulin levels. Less invasive, continuous monitoring enables the extraction and analysis of trends using AI, allowing predictive analytics to prevent emergencies by detecting hypo- and hyperglycemic events, diabetic ketoacidosis, or thyroid storms. One notable example is glucose detection utilizing near-infrared and RF sensing technologies combined with a random forest model for continuous glucose monitoring [54].

#### 4.2.6. Dermatology

Wireless sensing offers significant value in dermatology, particularly for monitoring skin moisture, quantifying itch, and evaluating wound healing and medication response. Kalasin et al. developed a smart bandage that uses RFID sensing and deep neural networks to monitor wound healing across three stages (inflammation, proliferation, and remodeling) after the application of corticosteroid cream [55].

#### 4.2.7. Nephrology

For tracking kidney function markers (i.e., creatinine and glomerular filtration rate) and managing fluid balance and dialysis, wireless sensing is a practical avenue. One such example is the study by Park et al., where hemodialysis patients with dysfunctional native arteriovenous fistulas were monitored. Shunt sounds before and after their percutaneous transluminal angioplasty were used to predict AVF stenosis with EfficientNetB5 and ResNet50 models [56].

### 4.3. Benefits and Limitations of Wireless Sensing Approaches

We have identified and evaluated several common benefits and limitations of AI-integrated wireless sensing approaches in their respective medical fields. One of the most frequently cited limitations in these studies was the small number of patients included for training and testing models and wireless sensing devices [27,29,30,33,35,36,38,48,49,53,54,56]. Limited study size, data collection methods, and variability in human activities posed challenges to the generalizability of many wireless sensing approaches [24,28,29,30,32,35,36,45,46,48,54,55,56]. Only a subset of surveyed papers included clinical validation of model predictions with commonly used or gold-standard assessments, such as polysomnography sleep studies [23,26,30,35,36,38,40,54,56]. While model performance overall correlated well with clinical assessments, limited inclusion of clinical validation in wireless sensing studies restricts real-world implementation. Additional limitations involved the influence of environmental factors and normal physiological processes on data recording. These factors affected both data quality and collection, with examples including breathing or movement impacting recordings, background noise reducing recording quality, and obstructions like furniture interfering with signal transmission [23,24,27,28,34,37,38,41,42,44,45,46,50,53]. There were also concerns regarding patient data privacy and security issues, including the possibility of an untrusted node in the network [31,39], along with the possibility of understanding the basis of AI predictions due to their “black box” nature [23]. Some other limitations included the limited compatibility of these systems due to software or operating systems [22] and the reliability of the communication channels due to signal degradation [25].

While these challenges are significant, several studies highlighted the notable benefits of their wireless sensing approaches in enhancing medical care. Most of these wireless sensors can be used to provide telehealth, improving healthcare access in areas where it is not possible to see a clinician as soon as possible or when there are mobility issues for the patient [22,24,31]. Due to the objective and automatic data collection techniques and the AI integration, some of these wireless sensing methods achieved high diagnostic and monitoring accuracy [22,23,24,29,30,32,33,36,41,56]. Furthermore, these approaches are non-invasive and safer avenues compared to the gold standard [23,30,37,38,39,40,41,54,56], allow for remote monitoring of conditions [23,34,54,55], and provide timely notification to improve clinical outcomes [25,29,44,47,49,52,53]. Wireless sensing additionally enables early detection, such as diagnosing MI in high-risk patients, improving clinical outcomes [27]. The ability to continuously monitor medical conditions or physiological parameters, along with the convenience of these sensing methods due to their low cost and non-invasive nature, were additional benefits highlighted in the studies.

### 4.4. Ethical Considerations

In addition to the technical and clinical considerations, ethical implications are also a significant focus in evaluating wireless sensing technologies due to patient privacy, informed consent, and data security issues. Van de Vel et al. highlighted concerns about patient privacy, given that their approach involves continuous video monitoring of patients, which poses risks to patient confidentiality and the security of patient medical information [29]. Collection, storage, and transmission of data are additionally vulnerable to security breaches and data leakage [31]. A few studies, however, addressed the privacy-preserving aspects in their designs, such as Zhang et al. and Wang et al. [45,53]. Such design elements underscore the necessity of building trust in these devices by embedding ethical safeguards in wireless sensing infrastructures and can play a role in the deployment of such technologies in medical settings.

## 5. Discussion

This systematic review of 34 research articles emphasizes the role of AI-integrated wireless sensing in healthcare, demonstrating its potential in real-time monitoring, diagnosis, and disease management. This review shows recent improvements and identifies several research gaps in the field, including limited sample size in studies, limitations of such technologies (i.e., battery life), lack of extensive clinical validation, and barriers in integration into clinical workflows. Furthermore, additional insights include shifting towards multimodal health monitoring and individualized patient algorithms as future directions for research. These points are discussed, with suggestions to improve these AI-based wireless sensing systems in healthcare, below.

### 5.1. Shift from Disease-Specific to Multimodal Monitoring

Most studies use wireless sensing to monitor specific conditions, such as Parkinson’s disease, MI, or sleep apnea. However, future systems could integrate data from multiple physiological parameters to create a more holistic picture of patient health. For instance, combining cardiopulmonary metrics with sleep patterns and movement data could enable earlier detection of complex health conditions, such as heart failure exacerbations or the onset of neurodegenerative diseases. This multimodal approach could be further enhanced by incorporating predictive AI algorithms to anticipate health events before they occur, moving healthcare towards a more preventive model.

### 5.2. Personalization for Improved Accuracy

Another well-suited area is the role of these models in individualized medicine. Most of the current AI systems rely on generalized algorithms, which may not account for individual variations in patient physiology or behavior. Personalization of these approaches—where models adapt to unique baseline data—could improve the accuracy of wireless sensing technologies. One such example is an AI system that learns the patient’s typical vital signs and offers more precise alerts for deviations that may indicate health risks. This individualized approach can improve trust in the technology by reducing false alarms and enhancing patient compliance.

### 5.3. Clinical Validation 

The above studies consistently point out the lack of extensive clinical validation as a major limitation. Along with expanding clinical trials, future work should facilitate rapid validation of these technologies and improve the deployment of AI technologies. One innovative approach could be decentralized clinical trials (DCTs) [63], which utilize telemedicine, remote monitoring, and digital tools to collect data from patients in real-world settings. DCTs could allow for more inclusive and diverse populations along with reducing the time and cost of validating studies across multiple sites. This would be particularly effective for testing wireless sensing devices in various environmental conditions, which is a major challenge we identified.

### 5.4. Integration with Healthcare Systems and Interoperability 

There is a need to address the lack of integration with existing healthcare systems. Most current wireless sensing technologies operate independently, which makes it hard for them to be integrated in clinical settings and with electronic health record (EHR) systems [64]. Future work should focus on developing interoperable systems that can integrate wireless sensing data with EHRs, allowing providers to access real-time patient data. Using AI to analyze these real-time data could additionally improve the decision-making process, reducing medical errors and enhancing healthcare delivery.

### 5.5. Sustainability and Device Lifespan

Due to battery life and device durability, sustainable and energy-harvesting technologies are potential avenues to extend device lifespan. Future research can explore the use of energy harvesting from ambient sources, including body heat, motion, and environmental light [65]. These advancements could lead to more autonomous and long-lasting solutions, which can be particularly helpful in low-resource settings where device recharging could be challenging.

### 5.6. Ethical Considerations and Data Privacy

Beyond technical limitations, ethical considerations regarding data ownership, privacy, and consent are becoming critical due to the increasing amounts of personal data that are being generated by these models. Current frameworks, including HIPAA, might not address the complexities of new developments in AI. A potential avenue for further research is blockchain-based solutions for securing patient data [66,67]. This can provide a tamper-proof record of data transactions, ensuring that patients can retain control over their health information while still allowing for data sharing for clinical use. Exploring such secure data-sharing approaches can significantly increase patient trust and help with the adaptation of wireless sensing technologies in healthcare.

### 5.7. Next-Generation Wireless Sensing Technologies

This review identifies radar, WiFi CSI, acoustic, and RFID as the primary wireless sensing technologies in healthcare, but next-generation technologies, such as quantum sensors [68,69] or 6G wireless networks [70,71], could offer new avenues for healthcare as well. Quantum sensors, for instance, could provide unprecedented high resolution and precision in detecting physiological signals at the molecular level, offering new applications in fields like cancer detection or monitoring of metabolic diseases. Similarly, the emergence of 6G networks, which promise ultra-low latency and high data throughput, could revolutionize real-time, continuous monitoring by enabling faster data transmission and more sophisticated AI algorithms that can operate in real time with minimal delays.

## 6. Conclusions

AI-driven wireless sensing technologies demonstrate promising potential in healthcare by providing real-time, non-invasive monitoring and early detection of medical conditions. However, to further improve the potential of these systems, future research must focus on developing more multimodal approaches, personalized algorithms, and extensive clinical validation. Addressing technical challenges such as background signal processing, environmental interference, device costs and sustainability, data privacy, and interoperability with existing systems is essential for facilitating widespread adoption and implementation into healthcare systems and patient homes; such challenges require further focused investigation to improve signal processing techniques and algorithms, device material and design, and health system software and data standardization. With advancements in next-generation wireless technologies and secure data-sharing techniques, AI-powered wireless sensing can reshape healthcare into a more personalized and accessible model.

## Figures and Tables

**Table 1:** Summary of wireless sensing techniques (adapted with permission from Wang and Shao 2022) [11].

	Sensing Techniques	Sensing Features	Pros	Cons
Radar	Continuous wave (CW), frequency-modulated continuous wave (FMCW), impulse radio ultra-wideband (IR-UWB)	Doppler shift Phase Distance	Large bandwidth; directional performance	High cost
CSI	WiFi orthogonal frequency division multiplexing (OFDM)	CSI amplitude CSI phase	High CSI resolution; ubiquitousness	Susceptible to environmental influence
RFID	CW	RFID phase	Directional performance; low cost	Channel hopping
Acoustic	CW FMCW OFDM	Acoustic phase Acoustic distance	High resolution	Susceptible to the environment; small coverage

**Table 2:** Study characteristics. DL = deep learning; ML = machine learning.

	Article	Year	Purpose	Level of Research Data	Wireless Sensing Type	Subject Number	Subject Type	AI Algorithm	Outcomes of the Model
Cardiopulmonary	Arjoune et al. [22]	2023	Detecting Still’s murmur and wheezes	Clinical validation	Acoustic	120+	Patients	DL	Still’s murmur—sensitivity 91.9%, specificity 92.6%, overall accuracy 92.2% Wheeze detection—sensitivity 83.7%, specificity 84.4%, overall accuracy 84.0%
	Howard-Quijano et al. [23]	2023	Measuring left ventricular ejection fraction	Clinical validation	Acoustic	81 (63 with cardiac pathology)	Patients and controls	DL and traditional ML	AUC 0.974 for detecting EF < 35% AUC 0.916 for detecting EF < 50%
	Lalouani et al. [24]	2022	Detecting breathing anomalies and COPD	Dataset analysis and conceptual validation	Acoustic	128 (64 with COPD) (from dataset)	Patients and controls	DL	Precision 0.97, recall 1.0, F1-score 0.98, accuracy 0.98 for patients with COPD (exact values not given, inferred from Figure 7 [24])
	Al-Momani and Garaibeh [25]	2014	Detecting and classifying asthma attacks	Clinical validation	Acoustic	18 patients (hospital); 144 controls (dataset)	Patients and controls	Traditional ML	Maximum probability of correct classification of 90% at signal-to-noise ratio (SNR) = 16 dB for SVM classifier and 86% at SNR = 17 dB for HMM classifier.
	Tseng et al. [26]	2016	Classifying normal and abnormal respiratory function	Clinical validation	Radar	50 (32 with “bad” respiratory function)	Participants with abnormal respiratory function and controls	Traditional ML	Classification accuracy 73.3%
	Zhang et al. [27]	2022	Detecting myocardial infarction	Clinical validation	Radar	60 (30 patients, 30 healthy)	Patients with controls	Traditional ML	Median detection accuracy of 66.5% when users are not stationary, and 81.2% when the users are stationary.
	Huang et al. [28]	2023	Diagnosing and prognosticating pediatric community-acquired pneumonia (CAP)	Clinical validation	Acoustic	198 (all with CAP)	Patients	DL	Subject-dependent setting: accuracy 97.3% for CAP diagnosis, 97.16% for CAP prognosis (sensitivity, specificity >96% for both diagnosis and prognosis) Subject-independent setting: accuracy 60.50% for CAP diagnosis, 42.18% for CAP prognosis (sensitivity, specificity >50% for CAP diagnosis and >39% for CAP prognosis)
Neurology/Psychology	Van de Vel et al. [29]	2016	Detecting tonicclonic and clonic seizures	Pilot patient study	Radar	2	Patients	Traditional ML	Mean sensitivity of 66.87% and false detection rate of 1.16/night.
	O’Brien et al. [30]	2021	Classifying dysphagia severity	Conceptual validation	Mechano-acoustic sensor	19 (9 patients and 10 controls)	Patients and controls	Traditional ML	Average predictive probability of 52.8% for mild severity, 53.8% for moderate severity.
	Verde et al. [31]	2019	Classifying healthy and pathological voices	Dataset analysis and conceptual validation	Acoustic	Combined voice sample datasets (796 healthy and 1207 pathological)		Traditional ML	Sensitivity 82.9%, specificity 86.2%, precision 85.7%, F-measure 84.3%, AUC 0.91, accuracy 84.5%
	Tahir et al. [32]	2019	Detecting Parkinson’s freezing of gait (FOG)	Clinical validation	WiFi CSI	15	Patients	DL	Highest accuracy of 99.7% for FOG detection; 94.3% for voluntary stop, 97.6% for walking slow
	Little et al. [33]	2021	Detecting speech as a marker of social functioning in late-life depression	Feasibility and validation study	Acoustic	58 (29 patients and 29 controls)	Patients and matched controls	DL	Sensitivity 94.6%, specificity 87.4%, 93.8% accuracy for speech detection Sensitivity 90.3%, specificity 86.2%, accuracy 89.95% for wearer vs non-wearer speech detection
Sleep Medicine	Mlynczak et al. [34]	2017	Classifying normal and snoring episodes	Conceptual validation	Acoustic	16	Healthy volunteers	DL	Accuracy 88.8%, Cohen’s kappa 0.7775, specificity 95.0%, sensitivity 76.8%, F1-score 82.4%
	Nguyen et al. [35]	2023	Monitoring sleep and producing auditory stimulation for sleep quality	Clinical validation and separate pilot patient study	Acoustic	377	Healthy volunteers	DL	Averaged accuracy of sleep scoring 84.08 ± 1.42% Strong correlation of 0.89 ± 0.03 with gold-standard PSG 87.8% agreement of sleep stage scoring with sleep technicians. Shortens duration of falling asleep by 24.1 min
	Kwon et al. [36]	2021	Classifying sleep stage	Clinical validation	Radar	65	Healthy volunteers	DL	Accuracy 82.6 ± 6.7%, Cohen’s kappa coefficient 0.73 ± 0.11
	Gu et al. [37]	2020	Monitoring sleep	Clinical validation	WiFi RSS and CSI	7	Healthy volunteers	Traditional ML	Short-term controlled experiments–detection accuracy 95.65%, false negative rate 2.16% 60 min real sleep studies–detection accuracy 98.22%, false negative rate 0%
	Ren et al. [38]	2019	Monitoring sleep and detect apnea	Conceptual validation	Acoustic	9	Healthy volunteers	Traditional ML	N/A (for sleep apnea). For different sleep events, TP around 80–90% and FP less than 10% (exact values are not given, inferred from Figure 16 [38]).
	Gui et al. [39]	2022	Monitoring sleep turnover activities and breathing rate	Conceptual validation	WiFi CSI	15	Healthy volunteers	DL	Mean accuracy 94.59% for turnover activities; 95.83% for sleep posture
	Yu et al. [40]	2021	Monitoring and classifying sleep stage	Clinical validation	WiFi CSI	12	Healthy volunteers	DL	Accuracy 81.8%
	Rossi et al. [41]	2023	Detecting sleep events	Conceptual validation	Acoustic	20	Healthy volunteers	DL	Classification accuracy of 97% for sleep apnea and 73% for snoring
Fall Detection	Torres et al. [42]	2017	Detecting bed and chair exits in hospital rooms	Clinical validation	RFID	26	Geriatric patients	Traditional ML	Overall recall 81.4%, precision 66.8% and F1-score 72.4%
	Taylor et al. [43]	2021	Classifying six human activities (walking, sitting, standing, picking up objects, drinking water, and falling)	Dataset analysis and conceptual validation	Radar	99 (from a dataset)	Healthy, elderly volunteers	DL and traditional ML	Accuracy 95.3% for the best performing model
	Garripoli et al. [44]	2015	Detecting real-time fall events and classifying movement	Conceptual validation	Radar	16	Healthy volunteers	Traditional ML	Sensitivity 100%, no false positives
	Wang et al. [45]	2022	Fall Detection	Conceptual validation	WiFi CSI	4	Healthy volunteers	Traditional ML	SVM—average classification accuracy 91.67% XGB—average classification accuracy 90.00%
	Wang et al. [46]	2017	Fall Detection	Conceptual validation	WiFi CSI	10	Healthy volunteers	Traditional ML	SVM: average detection precision 90%, average false alarm rate 15% Random forest—average detection precision 94%, average false alarm rate 13%
	Mercuri et al. [47]	2023	Detecting and localizing falls	Conceptual validation	Radar	6	Healthy volunteers	Traditional ML	No false positives or false negatives (TP: 40, FP: 0, TN: 117000, FN: 0) for fall detection Maximum mean absolute errors of 3.8 cm and maximum root-mean-square error of 7.5 cm (for measuring person’s absolute distance)
	Chu et al. [48]	2023	Fall Detection	Conceptual validation	WiFi CSI	22	Healthy volunteers	DL	Accuracy > 96% accuracy in all lab environments
	Ding and Wang [49]	2020	Fall Detection	Conceptual validation	WiFi CSI	10	Healthy volunteers	DL	Recognition accuracies of 90%, 91%, and 93% in indoor environments (laboratory, office, dormitory, respectively)
	He et al. [50]	2024	Fall Detection	Dataset analysis and conceptual validation	WiFi CSI	DARMS dataset (21 volunteers) [51]		Traditional ML	Accuracy of >95.25%
	Xia and Chong [52]	2023	Fall Detection	Conceptual validation	WiFi CSI	3	Healthy volunteers	DL	Accuracy, precision, and F1-score of 92% for detecting falls.
	Zhang et al. [53]	2023	Fall Detection	Conceptual validation	Radar	15	Healthy volunteers	Traditional ML	Recall 98.8%, precision 100%, false discovery rate (FDR) 0%, F1-score 0.994
Endocrinology	Sun et al. [54]	2023	Monitoring glucose levels	Clinical validation	RF near-infrared spectrometry	5	Healthy volunteers	Traditional ML	Root mean square error 21.06 mg/dL, mean absolute relative difference 7.31% for glucose prediction (compared to glucometer values).
Dermatology	Kalasin et al. [55]	2022	Classifying wound healing stages	Conceptual validation	RFID	10	Patients with inflamed skin	DL	Classification accuracy 94.6%
Nephrology	Park et al. [56]	2022	Predicting significant stenosis of arteriovenous fistulas	Clinical validation	Acoustic	40	Patients	DL	AUROC 0.98 for EfficientNetB5 and 0.99 for Resnet50 for predicting ≥50% AVF stenosis.
